# Sexual and Reproductive Health and Rights for Young Migrants in Sweden: An Ideal-Type Analysis Exploring Regional Variations of Accessible Documents

**DOI:** 10.3389/ijph.2024.1606568

**Published:** 2024-04-18

**Authors:** Albert Brunet Johansson, Anna-Karin Hurtig, Faustine Kyungu Nkulu Kalengayi

**Affiliations:** Department of Epidemiology and Global Health, Faculty of Medicine, Umeå University, Umeå, Sweden

**Keywords:** sexual and reproductive health, sexual and reproductive rights, migrant health, young adults, document analysis

## Abstract

**Objectives:** This study aims to map sexual and reproductive health and rights (SRHR) policies, strategies, and interventions targeting young migrants and describe the patterns of organisation, resources, and services across Sweden’s 21 regions.

**Methods:** We conducted a document analysis of accessible online documents on SRHR policies, strategies, and interventions targeting young migrants in Sweden’s 21 regions. We used ideal-type analysis of the documents to create a typology, which formed the basis of a ratings system illustrating variations in organisation, resources, and services across regions.

**Results:** Findings suggest that efforts aimed at addressing young migrants’ SRHR are fragmented and unequal across regions. While SRHR policies and strategies are commonplace, they routinely lack specificity. Available resources vary depending on region and resource type. Additionally, information and interventions, although common, do not consistently meet the specific needs of migrant youths.

**Conclusion:** This study suggests that fragmented efforts are fuelling geographic inequalities in fulfilling SRHR among young migrants. There is an urgent need to improve national coordination and collaboration between national and local actors in SRHR efforts targeting young migrants to ensure equity.

## Introduction

Sexual and reproductive health (SRH) is a fundamental right. This implies the right to the best possible standards of health related to sexuality and reproduction [1]. Fulfilling sexual and reproductive health and rights (SRHR) have significant payoffs: improved health and wellbeing, gender equality, and thriving communities, reflected in Sustainability Development Goals (SDGs) 3, 4 and 5 as well as in Swedish public health targets [1, 2].

Studies on migrant youth and SRHR are scarce. Existing research shows that conditions surrounding migration increase vulnerability to risky sexual behaviour and poor sexual health [3, 4]. Both young age and migration experience increase vulnerability to poor SRH outcomes. Evidence suggests that young migrants have special needs but limited access to available information and services in their destination countries [5, 6], who rarely meet their needs [3, 7]. A recent survey of migrants’ SRHR in Sweden indicated that young migrants often report poorer general and sexual health, but visit youth clinics less often, than their native-born peers. Migrant boys reported having experienced sexual violence more often than their native peers [8].

The Public Health Agency of Sweden (PHAS) has developed a national strategy for SRHR on behalf of the government with the goal of achieving good and equitable SRH for the entire population [9]. Recently a national action plan (NAP) was launched to guide national efforts on SRHR for the period 2023–2033 [10]. While SRHR work is nationally coordinated by the PHAS, it is implemented together with other national agencies, regional and municipal authorities, and stakeholders [9]. Sweden’s 21 regions are responsible for healthcare including sexual and reproductive care, which entails ensuring equal and accessible curative care, health promotion, prevention, and interventions in the field of SRH. This includes developing interventions targeting vulnerable groups prioritised in the NAP, such as older adults, people with disabilities, migrants, LGBTQ people, and youths and young adults [9, 10].

In Sweden, SRH services for young people are largely provided by youth clinics, a nationwide network developed in the 1970s to improve young people’s access to health information and services, particularly related to SRH [11]. However, youth clinics are not equally accessible to all, particularly socio-economically vulnerable groups, including young migrants, despite high needs [12, 13]. Various barriers to access have been identified, including language and cultural barriers, limited health literacy, and knowledge about rights and the Swedish health system [5, 14].

The Swedish Association of Local Authorities and Regions (SALAR) report that barriers to visiting youth clinics vary for different groups and across regions [12]. In other words, despite having a national SRHR strategy, the lack of effective national coordination remains a challenge. Instead of a cohesive approach, each region independently develops its own efforts to promote SRHR in collaboration with local authorities and stakeholders. So far, no study has examined the scope of regional SRHR resources addressing the needs of young migrants and how these vary across regions. Against this background, this study aims to map existing SRHR policies, strategies, and activities targeting young migrants in Sweden’s 21 regions to get an overview and describe variations across regions. This study will also serve as a baseline for future evaluation of the impact of the recently launched national action plan (NAP).

## Methods

### Study Design

We conducted a document analysis [15] examining accessible digital documents on SRHR policies, strategies and activities targeting young migrants in Sweden’s 21 regions to map and describe existing organisation, resources, and services. Using ideal-type analysis, we created a typology that formed the basis of a ratings system used to illustrate variations across regions [16].

### Data Types and Sources

This study uses the term document for any single record of information material, in line with Bowen’s definition of a document as something which “contain[s] text (words) and images that have been recorded without a researcher’s intervention” [15]. Sweden’s 21 regions host their own websites from which we selected freely available digital documents, all in Swedish, and all were sourced exclusively these websites. Our data set consisted of what Dalglish would categorise as official documents, implementation documents, legal documents, working documents, scholarly work, and media and communications [17].

### Data Search and Selection

A wide data set was gathered through key concepts, and then narrowed down through inclusion criteria before analysis. Data retrieval took place between December 2022 and January 2023, encompassing documents published before January 31, 2023. Key concepts and inclusion criteria were based on the concepts of migrants, youths, SRHR, and youth clinics. Youths were defined as adolescents and young adults in the ages of 16–29, who have been identified by the PHAS as a priority group in the context of SRHR [18]. We adopted the International Organization for Migration’s definition of the term migrant: “a person who moves away from their place of usual residence temporarily or permanently, and for a variety of reasons” [19]. Young migrants were defined as simultaneously belonging to these two groups.

The initial data set was selected using the following key concepts: a) child and youth health, b) sexual and reproductive health and/or rights, c) migrant health, d) migrant youth, and e) youth clinics. Data was gathered from regional websites through a thorough search of all available web pages relating to our key concepts, supplemented by use of internal website search functions and site maps. All available and accessible documents concerning one or more of our key concepts were selected, resulting in a large and diverse data set of 1,552 documents.

The data set was then narrowed down through the following inclusion criteria: documents were included if they directly or indirectly targeted a) youths, or b) migrants, or c) migrant youths; on issues related to i) SRHR, or ii) youth clinics. This resulted in a final data set of 657 documents. A list of the final data set can be found in Supplementary Material S1, and a visual representation of this process can be seen in Supplementary Material S2.

### Ideal-Type Analysis

We used ideal-type analysis (ITA) to analyse the 657 documents. ITA is a systematic methodological framework developed by Stapley et al for developing typologies from qualitative data [16]. A typology is an ordered set of categories, useful in organising and understanding phenomena according to their similarities and differences [20]. This methodology was adopted as it allowed systematic development of a typology describing efforts addressing young migrants’ SRHR based on qualitative documents. The ITA framework is a seven-step process involving: familiarization with the data set, writing case reconstructions, constructing ideal types, identifying optimal cases in the data, development of ideal-type descriptions, checking credibility, and making comparisons [16].

### Familiarization With the Data and Writing Case Reconstructions

Familiarization with the data allows the researcher to “gain a sense of the content and extent of the material collected” [16]. ABJ conducted detailed readings, organised the data, and took notes in the style of memos common in methodologies like grounded theory [21]. Thereafter, he wrote case reconstructions for each document as described by Stapley [16], i.e., “a written summary or description of the data for each participant that relates to the research question(s)” [16]. However, in this study, the equivalent unit of analysis was a single document, and the term document will be used in this study instead of “case” [16].

The case reconstructions focused on how each document related to our aims, as well as the form and function of the document such as perceived target audience, use case, content delivery method, et cetera. As such, case reconstructions were often substantially shorter than their source document, as information not relevant to our aims was disregarded in this step. Finally, every case reconstruction was reread alongside the original document, to ensure that no key information had been missed.

### Constructing the Ideal Types and Identifying Optimal Cases

Constructing ideal types entails systematically comparing all case reconstructions, letting the research questions define what patterns are highlighted by the researcher. This should result in all documents divided into groups based on their similarities, each document belonging to only one group [16]. ABJ constructed ideal types through an initial sorting of a subset of the data, namely, all documents from the (largest) Stockholm region. This initial typology was then used as a guiding template when sorting all remaining case reconstructions, refining it throughout the process. This method is recommended by Stapley when working with large data sets [22].

Debriefing sessions within the research team (ABJ, AKH, FKNK) were held regularly to develop the ideal types collaboratively. This resulted in 14 ideal types. To increase readability and contextualize our results we organised them into three themes: organisation (material related to strategy, management, and coordination of actors in the healthcare system), resources (material and knowledge available to service providers and professionals in the healthcare system), and services (services and information offered and made available to service users). This thematization is reflective of other analytical frameworks concerning health system functions [23].

One optimal case was then identified for each ideal type. The optimal case should “capture[s] the essence of that ideal type in a particularly pure or “optimal” form,” i.e., it should represent the essence of that ideal type. The purpose is to create an orientation point for the researcher in developing ideal type descriptions later [16]. This process was conducted by ABJ who systematically compared all documents to their corresponding ideal type and to each other. The resulting themes, ideal types and optimal cases can be seen in Table 1.

**TABLE 1:** Themes, ideal types, and optimal cases developed from documents concerning young migrants’ sexual and reproductive health and rights from Sweden’s 21 regions (Sexual and reproductive health and rights for young migrants in Sweden: an ideal-type analysis exploring regional variations of accessible documents, Sweden, 2023).

Theme	Ideal types	Optimal cases
Organisation	Collaborative organisations	Origo, organisation working with youth at risk for honour-related violence and related care practitioners and other professions
	Coordinators and networks	HIV/STI Mellansverige, a network promoting sexual and reproductive health in middle Sweden
	Policy and strategies	Västra Götaland’s region-wide strategy for sexual and reproductive rights and health
	Directives and missions	Framework agreement for joint operation of youth clinics in region Östergötland
Resources	Research and development	Region Stockholm’s centre for epidemiology and community health, and its specialised SRHR-unit
	Specialist resources	Una Norrbotten, specialist competencies team focusing on intimate partner violence
	Newsletters	Region Dalarna’s newsletter on sexually transmitted infections and HIV, published by the regional sexual health coordinator
	Knowledge resources	Series of studies on the effect of COVID-19 on sexual health in Stockholm by the centre for epidemiology and community health
	Professional development	LGBTQ-certifications for clinics, issued by civil society organisations representing LGBTQ-interests
	Operational development	Child impact assessment commissioned by region Stockholm regarding youth clinic reorganisation in the county
	Methods, tools, and materials	“SEXIT,” a methodology designed to help identify youth at risk of violence or sexual ill-health
Services	Information about health, rights, and related sources	Youth clinic online, UMO, and its translated equivalent, Youmo
	Information about available healthcare services	Search engines on regional websites or 1177 which find care facilities based on the user’s location
	Interventions	Healthcare communicators with specific cultural and language competencies working with migrant populations

### Forming Ideal-Type Descriptions

This step involved naming and developing descriptions of each ideal type, focusing on describing its core features. Using optimal cases as a baseline, this should result in clearly defined archetypes representing each ideal-type based on shared similarities [16]. ABJ wrote descriptions which were developed through debriefing sessions within the research team (ABJ, FNKN, AKH). The descriptions were then compared to the documents making up each ideal-type group and corresponding optimal case. The resulting ideal-type descriptions can be seen in Table 2.

**TABLE 2:** Themes, ideal types, and ideal-type descriptions developed from documents concerning young migrants’ sexual and reproductive health and rights from Sweden’s 21 regions (Sexual and reproductive health and rights for young migrants in Sweden: an ideal-type analysis exploring regional variations of accessible documents, Sweden, 2023).

Theme	Ideal types	Ideal-type descriptions
Organisation	Collaborative organisations	Collaboratively owned, managed, or funded independent organisations which produce, collate, and disseminate resources or interventions within a specific field. Often the result of collaboration between counties, municipalities, regions, and government agencies
	Coordinators and networks	Formalised collective (a group within an organisation or network connecting organisations) responsible for coordinating efforts within a specific field, including knowledge dissemination, event organising, and advocacy for their field. Unlike collaborative organisations, they do not form independent organisations
	Policy and strategies	Regulatory documents acting as operational guidelines on a regional scale by establishing strategies, long-term goals, or organisational values. The scope of these guidelines is wide, but they are abstract and inapplicable in day-to-day operations without proper operationalisation
	Directives and missions	Regulatory documents formalising operational requirements, goals, or responsibilities for specific units, actors, or sectors. Directives and missions have a narrow or clearly defined scope with relevance to day-to-day operations from a management perspective but lack clinical applicability
Resources	Research and development	Actor tasked with research and development within a field, which activities include knowledge production and dissemination, evaluation and development of tools and interventions, intelligence gathering
	Specialist resources	Units, task forces or other intra-regional actors with expertise in a specific subject area, who function as consultants, educators, and a source of skills development for other regional operations
	Newsletters	Bulletins, newsletters, and periodicals intended for healthcare practitioners informing on news, current affairs, events, or available courses within a certain field in a region
	Knowledge resources	General produced knowledge, such as scientific articles or reports, disseminated or produced by regions, but without clinical or operational applicability without further adaption
	Professional development	Educational efforts, such as online or offline courses, workshops, lectures, and seminars, undertaken to increase skills and competencies for healthcare professionals
	Operational development	Reports, evaluations, investigations, and follow-ups specifically and explicitly commissioned and designed to aid in operational development of regional operations
	Methods, tools, and materials	Clinical guidelines, methodological and practical tools, reference material designed for use by practitioners, and other aids with practical clinical applicability and use, and specifically designed with this in mind
Services	Information about health, rights, and related sources	Information intended for and accessible by the public on health, illness, and rights. This does not include information on healthcare facilities, practices, or availability
	Information about available healthcare services	Information about the availability, locations, procedures, or restrictions of healthcare services and facilities. This does not include general information on health and rights
	Interventions	Healthcare interventions targeting a group to promote health, prevent ill-health, and mitigate public health risk factors

### Checking Credibility

The framework as established by Stapley includes an independent researcher attempting to organise all “cases” into groups using the ideal-type descriptions developed earlier [16]. As described above, this step instead involved recurring debriefing sessions within the research team (ABJ, FKNK, AKH) whose disciplinary perspectives (sociology, medicine, migrant studies and health policy and systems research) contributed to the development of our typology. This collaborative process helped develop our ideal types and ideal-type descriptions and check credibility in accordance with Stapley’s framework, i.e., “…to assess the clarity of the ideal types developed by the original researcher…” and “…checking that the original researcher’s interpretations are grounded in the data” [16].

### Making Comparisons

The final step involves the write-up, including ideal-type descriptions, optimal cases, and comparing the documents in each ideal type to their corresponding optimal case [16]. To facilitate this step, a comparative ratings scale was adopted, focusing on a document’s degree of specificity in addressing young migrants’ SRHR. This was operationalised through a ratings system based on our inclusion criteria, ranging from one to three stars. The higher the number of stars, the greater the specificity.

1 star: document explicitly or implicitly addresses issues related to youth or migrant SRHR, or youth clinics.
2 stars: document implicitly addresses young migrants’ SRHR.
3 stars: document explicitly and specifically addresses young migrants’ SRHR.

At the end of this process, each document was assigned stars and sorted into region and ideal type. Regions were then compared based on their highest scoring document in each ideal type (see *Results* section).

## Results

The themes organisation, resources, and services are presented in three tables (Tables 4–6) displaying the ratings of each region by ideal types (categories of documents). The space has been left blank whenever no accessible document was found. Regions and ideal types are ordered according to their score: regions are ordered highest to lowest score based on the total number of stars in a row, and ideal types based on the total number of stars in a column. In summarising the results, emphasis was placed on the pattern of stars for each region by ideal type within each theme. See Table 3 for the number of documents sorted into each theme and ideal type.

**TABLE 3:** Number of analysed documents by ideal type for the organisation, resources, and services themes (Sexual and reproductive health and rights for young migrants in Sweden: an ideal-type analysis exploring regional variations of accessible documents, Sweden, 2023).

Theme	Ideal types	Number of documents
Organisation	Collaborative organisations	8
	Coordinators and networks	16
	Directives and missions	43
	Policy and strategies	23
	Total	90
Resources	Knowledge resources	28
	Methods, tools, and materials	206
	Newsletters	45
	Operational development	35
	Professional development	24
	Research and development	6
	Specialist resources	7
	Total	351
Services	Information about available healthcare services	106
	Information about health, rights, and related sources	71
	Interventions	39
	Total	216

### Organisation: Fragmented With Regional Disparities

The scoring patterns of the organisation theme, seen in Table 4, show that most regions had *directives and missions* documents. However, only Stockholm scored three stars, suggesting that this was the only region which had *directives and missions* specifically addressing young migrants’ SRHR. Instead, *directives and missions* in most regions were ambiguous and unspecific.

**TABLE 4:** Ideal-type ratings of documents concerning young migrants’ sexual and reproductive health and rights within the organisation theme across Sweden’s 21 regions (Sexual and reproductive health and rights for young migrants in Sweden: an ideal-type analysis exploring regional variations of accessible documents, Sweden, 2023).

Region	Directives and missions	Policies and strategies	Coordinators and networks	Collaborative organisations
Stockholm	★★★		★★★	★★★
Västernorrland	★	★★	★	★★
Västerbotten	★	★★		★★
Västra Götaland	★★	★	★	★
Dalarna	★★	★	★	
Gotland	★★	★	★	
Norrbotten	★	★		★★
Värmland	★★	★	★	
Västmanland	★★	★	★	
Gävleborg	★	★	★	
Jämtland Härjedalen	★			★★
Uppsala	★	★	★	
Blekinge		★	★	
Halland	★	★		
Jönköping	★★			
Kalmar	★	★		
Skåne		★		★
Sörmland	★		★	
Östergötland	★		★	
Kronoberg			★	
Örebro				

Similar patterns were observed for *policy and strategy* documents, although no region scored three stars, suggesting that none had implemented targeted policies and strategies. Documents for most regions instead suggested universal policies and strategies.

Only Stockholm scored a three-star rating for *coordinators and networks* and *collaborative organisations* documents. Most regions scored one star for *coordinators and networks*, while documents were unavailable for others. The same holds true for *collaborative organisations* in most regions, barring a few regions scoring two stars indicating documents implicitly addressing young migrants’ SRHR. An example of a two-star *collaborative organisation* is Normbanken, a collaboration for developing norm-critical sexual education material operated by the Västerbotten, Västernorrland, Norrbotten, and Jämtland Härjedalen regions. Normbanken accounts for all two-star ratings in *collaborative organisations*.

### Resources: Scattered and Unequally Distributed

As indicated in Table 5, the scoring patterns of the “resources” theme reveal unevenly distributed resources across regions. *Methods, tools, and materials* documents were available in all regions except one, consistently scoring two or more stars. A few even scored three stars, indicating *methods, tools, and materials* that specifically addressed young migrants’ SRHR. The commonly used visual communication aids developed by the Västra Götaland region, DART, for example, includes material specifically addressing young migrants’ SRHR. This popular tool may therefore contribute to higher ratings of documents of this type in multiple regions. Regions often adopted clinical guidelines specifically addressing young migrants on SRHR issues such as female genital mutilation (FGM), honour-related violence and oppression (HRV), and intimate partner violence (IPV), contributing to higher ratings. Notably, documents concerning FGM, HRV and IPV were much more common within this theme than in organisation or services. This was especially true for *methods, tools, and materials*, where a majority of documents concerned FGM, HRV, or IPV.

**TABLE 5:** Ideal-type ratings of documents concerning young migrants’ sexual and reproductive health and rights within the resources theme across Sweden’s 21 regions (Sexual and reproductive health and rights for young migrants in Sweden: an ideal-type analysis exploring regional variations of accessible documents, Sweden, 2023).

Region	Methods, tools, and materials	Operational development	Professional development	Knowledge resources	Newsletters	Specialist resources	Research and development
Stockholm	★★★	★★★	★★★	★★★	★★	★★★	★
Västra Götaland	★★★	★★★	★★	★★★	★★	★★	★
Dalarna	★★	★★		★★	★★		
Jämtland Härjedalen	★★★	★	★★	★			
Norrbotten	★★★	★		★		★★	
Gotland	★★	★	★★★				
Skåne	★★	★		★★			★
Sörmland	★★		★		★★	★	
Östergötland	★★	★★	★	★			
Västerbotten	★★★	★		★			
Västmanland	★★	★★	★				
Blekinge	★★	★★					
Kronoberg	★★		★	★			
Uppsala	★★				★★		
Värmland	★★		★			★	
Västernorrland	★★★		★				
Gävleborg	★★			★			
Halland	★★		★				
Jönköping	★★		★				
Kalmar	★★						
Örebro		★		★			

Documents pertaining to *operational development*, *professional development, knowledge resources, newsletters, specialist resources*, and *research and development* exhibited erratic patterns. Resource documents were either unavailable or inaccessible for at least three of the seven categories, except for the Stockholm and Västra Götaland regions. These were also the only regions scoring at least two or three stars on all resource categories except *research and development*, suggesting they had resources which implicitly or explicitly addressed young migrants’ SRHR in all but this category. Finally, only three regions (Stockholm, Västra Götaland, and Skåne) had *research and development* documents. They all scored one star, suggesting documents did not specifically address young migrants’ SRHR.

### Services: Divergent Patterns Across Regions

In Table 6, the results reveal divergent patterns across the three categories of the “services” theme. All but two regions had documents pertaining to *interventions*. However, only a few regions scored three stars, specifically addressing young migrants’ SRHR, for example, through adapted communication and education interventions targeting migrant youths. Most regions scored either one or two stars, often through universal interventions such as making youth clinics digitally available and accessible.

**TABLE 6:** Ideal-type ratings of documents concerning young migrants’ sexual and reproductive health and rights within the services theme across Sweden’s 21 regions (Sexual and reproductive health and rights for young migrants in Sweden: an ideal-type analysis exploring regional variations of accessible documents, Sweden, 2023).

Region	Interventions	Information about available healthcare services	Information about health, rights, and related sources
Stockholm	★★	★★★	★★★
Jämtland Härjedalen	★★	★★	★★★
Norrbotten	★★	★★	★★★
Västra Götaland	★★	★★★	★
Västerbotten	★	★	★★★
Västmanland	★★	★	★★
Örebro län	★	★★	★
Gävleborg	★★★	★	
Värmland	★★	★	★
Västernorrland		★	★★★
Blekinge	★★	★	
Dalarna	★	★	
Kronoberg	★★★		
Skåne	★★	★	
Sörmland	★★★	★	
Gotland		★★	
Halland	★	★	
Uppsala	★		★
Östergötland	★	★	
Jönköping	★		
Kalmar	★		

Likewise, almost all regions had accessible *information about available healthcare services* documents. Several regions received a one-star rating, suggesting that they had generic or standardised information targeting the general population. For instance, many regions referred to content on 1177.se, Sweden’s national health and healthcare information system. Some scored two stars, but only the Stockholm and Västra Götaland regions scored three stars. The Stockholm region’s youth clinic website, for example, was available in multiple languages.

There were no available documents related to *information about health, rights, and related sources* for several regions. Only a few regions scored three stars (Stockholm, and the four northernmost regions Jämtland Härjedalen, Västernorrland, Västerbotten, and Norrbotten), suggesting that very few regions had information specifically targeting and addressing young migrants’ SRHR. Stockholm’s three-star rating, for example, was mainly based on documents from Origo, an advocacy organisation for victims of HRV, child marriage, and similar issues. The three-star ratings of the northernmost regions were based on sex-ed material for young migrants from Normbanken, mentioned earlier.

## Discussion

Results show that efforts to fulfil young migrants’ SRHR are disparate across Sweden’s 21 regions, suggesting that fragmented efforts are fuelling geographic inequalities in SRHR among young migrants as their opportunities to fulfil these rights might vary depending on their place of residence. Analysis of documents reveal regional disparities in organisation, unequally distributed resources, and divergent patterns in availability and accessibility of services. These disparities can be partially attributed to the decentralized nature of the Swedish health system, which delegates primary responsibilities to regions and municipalities [24]. The SALAR mapping report of youth clinics has also suggested that barriers for young people to visit youth clinics vary across both locations and groups [12]. The NAP on SRHR for 2023–2033 is expected to address some of these challenges, but risks falling short as it fails to explicitly address young migrants as a priority group, despite their intersectional vulnerability.

### (Un)common Types of Documents and Their Relevance

Documents categorised as *methods, tools, and materials*, *interventions*, *information about available healthcare services*, or *directives and missions* were available in almost all regions. Common to these documents is their relevance to daily practice, making them essential to policymakers and health professionals. In particular, *methods, tools, and materials* documents generally either implicitly or specifically targeted young migrants’ SRHR, which might reflect efforts to improve practitioners’ cultural competency. Health practitioners have previously identified lack of cultural competency, knowledge and skills as potential barriers in their encounters with migrant patients [25, 26]. Access to continued education and professional development for professionals is included in one of the NAP’s prioritised areas [10].

Similar patterns were observed with *intervention* documents, although they lacked the consistent specificity of *methods, tools, and materials* documents. Some regions have adopted universal approaches while others opted for targeted interventions. These diverse approaches may lead to inequalities in fulfilling SRHR among young migrants as targeted interventions may be more effective in meeting their specific needs [27]. The NAP for SRHR also recommends adapting interventions to reach prioritized groups, including migrants and young people, while employing an intersectional approach to target and reach vulnerable sub-groups within these larger groups [10].

*Information about available healthcare services* and *directives and missions* documents were less common, with generally lower and more varied specificity, suggesting that they are not specifically targeting young migrants’ SRHR. Health professionals in a study by Thomée et al. (2016) highlighted organizational challenges in terms of weak and unclear directives, as well as local and regional diversity [26]. Similarly, lack of clarity and disparities in mission statements have also been reported in the SALAR mapping [12]. The NAP recommends integrating SRHR into relevant governance and policy documents, alongside its aims to enhance coordination within the field. It emphasizes a compensatory perspective, which entails providing targeted support and development to groups with greatest needs [10].

Several, but not all, regions also had documents pertaining to *operational development*, *professional development*, *knowledge resources*, *information about health, rights, and related sources*, *policies and strategies*, and *coordinators and networks*. In contrast to common categories, these documents were more abstract and related to organisational and human resource aspects and showed dissimilar patterns regarding specificity. Several regions had *information about health, rights, and related sources* documents that specifically targeted young migrants’ SRHR, but there were quite few for *operational development*, *professional development,* and *knowledge resources*, suggesting a lack of these in several regions. The SALAR report has stressed the need to increase access to knowledge support and strengthen knowledge exchange between youth clinics [12]. It is possible that many regions are relying on resources produced within academia or at the national level by the PHAS or other agencies. For instance, the Swedish Agency for Youth and Civil Society, MUCF, is a government agency which produces and disseminates knowledge and organises workshops and training sessions for different stakeholders involved at the regional level [28]. However, these resources may not effectively address contextual factors. Enhancing knowledge production and monitoring related to SRHR is one of the six priority areas identified in the NAP [10].

Wherever available, *coordinators and networks* and *policy and strategies* documents almost exclusively score one star, suggesting insufficient specificity in addressing young migrants’ SRHR. Regions may have mainly opted for universal policies which may be ineffective in addressing the specific needs of young migrants. Health professionals in Tirado et al’s (2022) study mentions a lack of required structure to build dependable SRH services for young migrants that go beyond one-time interventions [14]. The NAP recommends integrating SRHR initiatives into public health programs [10].

Finally, *collaborative organisations*, *newsletters, specialist resources*, and *research and development* documents were rarely available. This scarcity could partly be explained by lack of coordination, insufficient resources, low prioritisation, or that related activities are not included in regional missions. Most worrisome is that only Stockholm had available documents on *collaborative organisations* specifically addressing young migrants’ SRHR. The SALAR report also revealed that collaboration between youth clinics and other actors takes place to varying degrees in different regions [12]. Collaboration is important as all policy sectors play a role in healthy public policy [27]. Tirado et al (2022) have stressed that implementing strategies to improve SRH services may require involving both regional and national decision-makers and multi-stakeholders like communities, civil society, and young migrants themselves [29].

*Newsletters* and *specialist resources* had similar ratings but were even rarer. *Research and development* documents were only available in densely populated regions (Stockholm, Västra Götaland, and Skåne), which are also the regions with the highest number of migrants [30], although they did not specifically address young migrants’ SRHR, suggesting lack or insufficient production and dissemination of relevant knowledge. Strengthening knowledge production and monitoring in the SRHR field is another priority area mentioned in the NAP [10].

### Culture-Associated Topics: Pertinent, but May Reinforce Cultural Stigma and Othering

Documents commonly addressed topics like FGM, HRV, and IPV while specifically targeting young migrants, leading to documents on these topics receiving high ratings, especially *methods, tools, and materials*. While these topics are pertinent concerns for this group, exclusively focusing on them within the context of SRHR could inadvertently reinforce cultural stigma associated with these issues and contribute to a sense of otherness. It is essential to recognize that these challenges occur across all cultures and settings. Amroussia et al’s (2022) study emphasises the importance of avoiding othering young migrants in discourse surrounding HRV and oppression [25].

### Strengths and Limitations

To our knowledge, this represents the first comprehensive mapping study of accessible digital documents on SRHR policies, strategies and activities specifically targeting young migrants across Sweden’s 21 regions. This study sheds light on regional disparities which may lead to inequalities in young migrants’ ability to fulfil their SRHR needs. Additionally, it demonstrates how the ideal-type analysis framework can be effectively used in health system research to create descriptive typologies that highlight disparities across different regions within a country.

Document analysis has both strengths and limitations. One of its strengths is that documents provide a rich source of data offering insights into SRHR-policies, strategies and interventions targeting young migrants. Additionally, this method is less intrusive, reducing biases inherent in face-to-face interactions common in surveys and interviews. By gathering diverse types of data, we were able to approach the topic from multiple perspectives and sources. In addition, we have provided detailed information about the research process and used regular debriefing sessions to enhance credibility.

However, there are potential limitations. Ensuring authenticity and reliability of documents can be challenging. In our study, we relied on documents from official sources and selected through specific criteria. Yet, some documents lacked valuable information (e.g., some documents implicitly target young migrants), limiting the depth and scope of our analysis. Additionally, the accessibility of relevant documents varied, potentially affecting how well our study reflects real conditions in different regions. Furthermore, the “digital landscape” from which we sourced documents is in constant flux, impacting retrievability. Documents are constantly added, removed, made (in)accessible, or updated making our analysis a momentary snapshot of a dynamic system. Different analysts may interpret the same documents differently, although the authors’ good understanding of the Swedish context and the studied topic mitigates this variability. Considering this, it is crucial to interpret our results with caution and view them as a foundation for further efforts.

It is necessary to restate that our goal was to map currently existing efforts. It is therefore beyond the scope of the study to assess how this correlates with access to high quality SRH services. Additionally, this study did not explore geographic and demographic factors that may contribute to our findings, such as the size and proportion of young migrant populations across regions. Further research is warranted. However, our analysis is grounded in rich material with extensive geographical coverage resulting from a comprehensive data search. This robust dataset has enabled us to illuminate patterns of disparity across organisation, resources, and services on a broad scale.

### Conclusion

This study suggests that fragmented regional efforts contribute to geographical inequalities in meeting SRHR needs among young migrants. This study identified actionable areas that align with the priorities of the NAP, including: improving national coordination of SRHR efforts targeting young migrants; enhancing collaboration between national and regional actors; and establishing clear directives and developing specific goals and strategies to address young migrants’ SRHR needs. Additionally, adapting services and information to reach young migrants, involving migrant communities in development and implementation of SRHR programs, and strengthening knowledge production and evidence-based policymaking across regions are crucial steps.

However, it is important to note that young migrants, who simultaneously belong to multiple prioritised groups, are not explicitly mentioned in the NAP. Therefore, emphasising an intersectional perspective when working with these groups becomes essential. Additionally, it is worth noting that while the NAP serves as a guiding (non-legal) framework, regional autonomy takes precedence. Local decision-makers, therefore, have a crucial role in translating the NAP into action. This process requires direction from regional political leadership and allocation of resources for effective implementation. Further research is warranted to explore the drivers and consequences of these disparities and their impact on access to and quality of services.
