# Supplementary material for: Sexual and Reproductive Health and Rights for Young Migrants in Sweden: An Ideal-Type Analysis Exploring Regional Variations of Accessible Documents
Source: Int J Public Health. 2024 Apr 18;69:1606568. doi: 10.3389/ijph.2024.1606568 (PMC11063276; doi:10.3389/ijph.2024.1606568)
Supplement: Supplementary file 1 [file DataSheet2.pdf]

Supplementary file 2. Chart of data selection process including initial data selection through key concepts and data screening using inclusion criteria (Sexual and reproductive health and rights for young migrants in Sweden: an ideal-type analysis exploring regional variations of accessible documents, Sweden, 2023).

	Initial data collection - key concepts -	Data screening - inclusion criteria -
Region Blekinge	31 documents	13 documents
Region Dalarna	32 documents	24 documents
Region Gotland	36 documents	25 documents
Region Gävleborg	45 documents	18 documents
Region Halland	41 documents	29 documents
Region Jämtland Härjedalen	59 documents	27 documents
Region Jönköpings län	48 documents	14 documents
Region Kalmar län	54 documents	15 documents
Region Kronoberg	69 documents	22 documents
Region Norrbotten	95 documents	45 documents
Region Skåne	89 documents	27 documents
Region Stockholm	232 documents	137 documents
Region Sörmland	61 documents	22 documents
Region Uppsala	47 documents	21 documents
Region Värmland	77 documents	24 documents
Region Västerbotten*	67 documents	19 documents
Region Västernorrland	53 documents	10 documents
Region Västmanland	54 documents	21 documents
Region Örebro län	39 documents	6 documents
Region Östergötland	52 documents	21 documents
Västra götalandsregionen	271 documents	117 documents
**Total data set**	**1552 documents**	**657 documents**

*Documents sourced from Normbanken (a website shared between the northern regions of Jämtland Härjedalen, Norrbotten, Västerbotten, and Västernorrland) are included in Region Västerbotten
